# 

**Published:** 1893-09-02

**Authors:** 


PRICE TWOPENCE.

No. 362, Vol. XVI.—Sept. 2nd, 1893.

Registered at the General Post Office as a Newspaper for transmission in the United Kingdom and Abroad.]

SEPTEMBER 2ND,

WITH EXTRA NURSING SUPPLEMENT.

Per Annum, 8s. 6d., post free.

Monthly Parts, 6d.; post free, 8d.

Ditto per Annum, 8s., post free.

### THE HOSPITAL

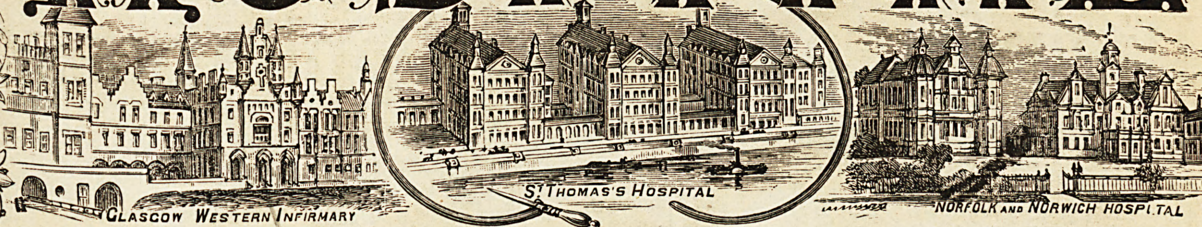

WITH EXTRA NURSING SUPPLEMENT.

No. 362. Vol. XIV. SATURDAY, SEPTEMBER 2ND, 1893. [TWOPENCE.

